# Factors Associated with Referral to Low Vision for Patients with Advanced Glaucoma

**DOI:** 10.3390/life16010012

**Published:** 2025-12-22

**Authors:** Julia Ernst, Janice Huang, Jakob Tsosie, David J. Ramsey

**Affiliations:** 1 Department of Ophthalmology, Tufts University School of Medicine—Lahey School of Medicine, 31 Burlington Mall Rd, Burlington, MA 01805, USA; 2 Division of Ophthalmology, Department of Surgery, UMass Chan—Lahey School of Medicine, 31 Burlington Mall Rd, Burlington, MA 01805, USA; 3 Brandeis University, Waltham, MA 02453, USA; 4 Department of Biomedical Sciences and Disease, New England College of Optometry, 424 Beacon St, Boston, MA 02115, USA

**Keywords:** low vision, glaucoma, low vision referrals, low vision services, vision rehabilitation

## Abstract

Glaucoma is one of the most common causes of irreversible visual impairment world wide. Providing low vision rehabilitation (LVR) services is a primary mode of support for patients with permanent vision loss. This retrospective, cross-sectional study evaluated the rate at which patients with severe open-angle glaucoma (OAG) were referred for LVR services at an academic medical center. Patient demographics, glaucoma severity, appointment history, performance on visual field (VF) testing, presenting visual acuity (VA), and change in best-corrected visual acuity (BCVA) after low vision refraction were abstracted from the electronic record and summarized by using descriptive statistics. Logistic regression analysis was used to assess the relationship between study variables and the likelihood of referral for LVR evaluation. Out of 522 patients with severe OAG, 88% of whom qualified as having low vision, 14 were referred for an LVR evaluation (2.7%). Referrals were most strongly associated with VA (adjusted odds ratio [aOR], 7.20; 95% confidence interval [CI], 2.11–24.64, *p* = 0.001) but not glaucoma-associated VF loss (aOR, 0.90; 95% CI, 0.24–3.37, *p* = 0.876). Thirteen of 14 patients referred for LVR completed visits (93%). More than one-third of those patients improved in their better-seeing eye after a low vision refraction, gaining an average of −0.18 ± 0.24 logMAR (half gaining ≥2-lines of BCVA). Patients with severe OAG are at risk of progressive visual disability from their eye disease. We found, however, that the majority of these patients were not referred to LVR services, despite meeting eligibility criteria and growing evidence demonstrating their potential benefit.

## 1. Introduction

Glaucoma is the second leading cause of irreversible blindness in the world [1]. Open-angle glaucoma (OAG) is a multifactorial, degenerative and progressive optic neuropathy often associated with elevated eye pressure leading to visual field (VF) defects, which may involve central vision [2]. Vision loss from glaucoma can profoundly impact an affected individual’s quality of life, even at early stages of the disease [3]. Research has shown that individuals with advanced stages of the disease experience reduced mobility, slower reading speeds, and often have difficulties performing activities of daily living [4,5,6]. Glaucoma also is associated with reductions in contrast sensitivity and dark adaptation [7,8]. As the world population grows and the average age increases, the expected number of cases of vision impairment from OAG is expected to increase and the cost of treating affected individuals is forecasted to more than double by 2050 [9,10].

Vision loss from glaucoma may occur because of progressive degradation of the VF or may be a consequence of compromise of central vision, i.e., from a vision-impairing central scotoma [2]. Patients with glaucoma may also experience low vision, which is defined as sight that cannot be corrected with refraction or surgery. The World Health Organization (WHO) defines low vision as having a visual acuity (VA) with best possible correction worse than 20/70 in the better-seeing eye or a VF of less than 20 degrees in the greatest VF diameter [11]. In the United States approximately 7 million individuals have varying degrees of low vision and 1 million are blind [12]. Globally, it is estimated that around 237 million people have low vision and 38 million are blind [13]. According to a survey on the importance of eye health in the US population, loss of sight was rated as having the greatest effect on daily living, significantly worse than loss of memory, hearing, or speech [14]. In patients with glaucoma, a loss of VF is often more disabling than loss of central visual acuity [3]. Glaucoma patients commonly report reduced mobility, leading to activity limitations and hence reduced socio-emotional wellbeing as the major adverse effects of their condition [4,6].

Vision rehabilitation is the primary treatment option for patients with irreversible vision impairment and low vision. The American Academy of Ophthalmology Preferred Practice Pattern Vision Rehabilitation Committee recommends that patients with VA worse than 20/40, contrast sensitivity loss, or peripheral, or a central scotoma should be referred for low vision evaluation [15]. LVR services include an eye examination and visual function assessment, and often include a psychological assessment, employment counseling, driving fitness evaluation, and necessary follow-up care may even take place in a patient’s home [16]. Vision rehabilitation comprises a variety of individualized treatment options including interventions to improve mobility, orientation, eccentric viewing, assistive technology assessment and use of optical aids [17]. There is strong evidence supporting the effectiveness of these services. For example, nearly half of patients showed clinically meaningful improvements in visual function after receiving LVR services, with improvements in patient mobility, visual information processing, and motor skills [18,19,20].

Despite evidence supporting the effectiveness of LVR and their growing availability, the utilization of LVR in the US remains low [3]. This is especially troubling given the enormous psychosocial and economic burden imposed by glaucoma [9,10]. Research into the rate and barriers to the referral of patients with glaucoma to LVR services is limited [16,21]. Current research indicates that there are barriers both at the physician and patient level. The goal of this study was to evaluate the rate at which patients with severe OAG were referred for LVR services at an academic medical center and to determine key patient characteristics associated with referrals.

## 2. Materials and Methods

The research followed the tenets of the Declaration of Helsinki and the Strengthening the Reporting of Observational Studies in Epidemiology reporting guidelines (https://www.equator-network.org/reporting-guidelines/strobe/, accessed on 1 March 2022) (Supplemental Figure S1) [22]. The requirement for informed consent was waived because of the retrospective nature of the study, and a waiver was granted by the Institutional Review Board of the Lahey Hospital & Medical Center, Burlington, MA, USA. Data were gathered and secured in compliance with the Health Insurance Portability and Accountability Act.

### 2.1. Study Participants

This study comprised a retrospective cross-sectional analysis of patients with advanced OAG spanning a 12-month period (1 March 2021 to 28 February 2022) that was completed as part of a quality improvement project at Lahey Hospital & Medical Center (Burlington, MA, USA). Patients were identified based upon International Classification of Diseases, Tenth Revision, Clinical Modification (ICD-10 CM codes) and included those patients with severe primary open-angle glaucoma (H40.11X3), low-tension glaucoma (H40.12X3), pigmentary glaucoma (H40.13X3), and pseudoexfoliation glaucoma (H40.14X3) involving one or both eyes. Other specified types of glaucoma, including neovascular glaucoma (H40.89) and narrow-angle glaucoma (H40.2), were excluded from this study. Also excluded were those patients who had significant lens or other media opacities, recent ocular surgery, amblyopia, or trauma.

### 2.2. Patient Characteristics

Patient characteristics were compared between those who received a referral to LVR services and those for whom a referral was not documented. Demographic data (age, sex, race, ethnicity, primary language spoken, and distance from the eye clinic), class of health insurance, and most recent clinical characteristics (VA, ophthalmology appointment data) were extracted from the electronic health record (EHR) by means of a custom reporting tool [23]. A Microsoft^®^ Excel^®^ 2016 Visual Basic for Applications (VBA) program (version 16.0; Microsoft Corporation, Redmond, WA, USA) was used to access Microsoft Maps (Microsoft^®^ Maps, Version 1611), which calculated the number of miles between each patient’s home and the clinic by zip code. Clinical characteristics including glaucoma severity, VA, and visual impartment status were documented for every patient.

Patients whose BCVA in the better-seeing eye was between 20/40 and 20/70, inclusive of these endpoints, were classified as having “mild” visual impairment, whereas those with a BCVA below 20/70 or less than 20 degrees of uninterrupted horizontal VF around the point of fixation were considered to have low vision for the purposes of our study [15]. Since no patients had undergone Goldman Visual Field testing, we relied upon the most recent Humphrey Visual Field (HVF) test results within the study period. If multiple HVFs were performed, preference was given to the 24-2, then 30-2, and finally 10-2. A reliable VF was determined by the following indices: fixation losses < 20%, false negative < 20%, and false positive < 20%. Using the raw sensitivity data plot, we recorded the visual sensitivity in decibels (dB) of the central, horizontal 20 degrees (10 degrees temporally, 10 degrees nasally) around central fixation, both above and below the horizontal meridian, which enabled uniform classification of VF defects across different VF types. We then calculated the greatest, uninterrupted horizontal VF. Sensitivity values of 10 dB on the data plot were considered to be areas of VF loss, as this corresponds to a significant visual field defect, equal to 1000-fold reduction from the maximum, especially if present in the central visual field [24]. Finally, legal blindness as defined in the U.S.A. was set according to the U.S. Social Security Administration regulation (20 CFR § 404.1581) as either having a BCVA ≤ 20/200 in the better-seeing eye or a VF of less than 20 degrees around central fixation [24]; and the subset of patients whose worse-seeing eye had a BCVA ≤ 20/200 and whose better-seeing eye had a BCVA ≤ 20/70 were considered functionally monocular [15]. All definitions for low vision aligned with the categories established by the 2019 WHO report on vision [11].

### 2.3. Low Vision Rehabilitation Utilization

Referrals for LVR services and outcomes of completed consultations, were abstracted from the EHR for each patient. For patients who attended an LVR consultation, treatment effectiveness was determined by comparing the BCVA for each eye at the time of referral with the BCVA achieved after distance refraction by a low vision specialist. Any change in low vision category or monocular status was also noted. In addition, patients who received magnifiers or mobility training were noted.

### 2.4. Statistical Analysis

Data were analyzed using SPSS (version 28.0, IBM, Armonk, NY, USA). Snellen visual acuity (VA) in the better- and worse-seeing eyes were converted to the logarithm of the minimum angle of resolution (LogMAR). Continuous variables were assessed for normality using the Shapiro–Wilk test. For normally distributed variables, data are presented as mean (±SD) and significance evaluated by means of Student’s *t*-test. For non-normally distributed variables, data are presented as median with interquartile range [IQR] and significance evaluated by means of the Mann–Whitney U test. Frequency (count) and relative frequency (percentage) were employed for categorical data, and the Chi-square test was used to assess the difference between categorical variables. Change in BCVA and low vision status by category after low vision refraction was assessed by using the Wilcoxon Signed-Rank Test or Sign Test for categories that included fewer than ten cases, as separate paired analyses. Pearson’s correlation coefficient was used to assess for any association between continuous variables. Logistic regression analysis was used to assess the relationship between study variables and the likelihood of referral for LVR evaluation. Variables with statistically significant effects were chosen for inclusion in the final model. Adjusted odds ratios (aORs) and 95% confidence intervals (CIs) were calculated for each variable. All tests were two-sided, and *p*-values below 0.05 were considered statistically significant.

## 3. Results

Out of 522 patients identified with severe OAG, 88% (460 patients) qualified as having low vision (Table 1). A total of 14 patients out of these 522 patients were referred for an LVR evaluation in the study timeframe. This constitutes 2.7% of the total patient with severe OAG. The majority of these patients (93%) completed LVR visits.

Patients who were referred tended to be older (87 vs. 79 years old, *p* = 0.005) (Table 1). By contrast, sex, race, ethnicity, primary language spoken, type of health insurance, employment status, or distance from the eye clinic were not associated with the likelihood of referral for LVR services. Similarly, severe glaucoma in one or both eyes, a history of glaucoma treatment with laser or surgery, and prior cataract surgery—despite its potential to improve visual function—had no influence on the likelihood of being referred. By contrast, visual acuity in the better-seeing eye (0.704 ± 0.365 logMAR) and also worse-seeing eye (1.763 ± 0.836 logMAR) was associated with LVR referral. Similarly, low vision status (43%, *p* = 0.001) and legal blindness (43%, *p* = 0.001) based on VA were associated with LVR referral but not when based on VF (low vision status 79%, *p* = 0.171; legal blindness 21%, *p* = 0.101).

After controlling for age, which had a modest impact on whether a patient was referred for LVR, we assessed if VA or VF loss, or both influenced referral rate (Table 2). We found that low vision status defined by VA was the factor most closely associated with referral for LVR (aOR, 7.20; 95% confidence interval [CI], 2.11–24.64, *p* = 0.001). By contrast, low vision status defined on the basis of VF loss was not associated with referral for LVR services (aOR, 0.90; 95% CI, 0.24–3.37, *p* = 0.876). Of note, 484 patients (92.7%) had VF-testing results. A similar analysis found that VA in the worse-seeing eye (aOR, 2.03; 95% CI, 1.08–3.80, *p* = 0.027) but not the better-seeing eye (aOR, 1.76; 95% CI, 0.68–4.57, *p* = 0.245) was associated with referral for LVR. A similar quantitative analysis could not be performed for any specific visual field parameter because of variation in VF type and protocol across patients. Factors excluded from our model because they did not impact referral included sex, race, ethnicity, primary language spoken, type of health insurance coverage, employment status, distance from the eye clinic, glaucoma severity, treatment characteristics, and lens or monocular status.

Out of the 14 patients who were referred for an LVR evaluation, 13 met criteria for having low vision. An examination of underlying factors associated with low vision found that six of those patients identified with low vision met criteria for low vision when assessed solely based on VA (43%). By contrast, 11 patients (85%) met criteria for low vision based upon their degree of VF loss. Finally, four patients (31%) met criteria for low vision based on both their visual acuity and VF. Among all patients who met VA-based low vision criteria in the full cohort, 6 of 14 referred patients (43%) and 36 of 508 non-referred patients (7.1%) qualified based on VA, indicating that VA-defined low vision was strongly associated with referral (*p* < 0.001). In contrast, among all patients who met VF-based low vision criteria, 11 of 14 referred patients (79%) and 431 of 508 non-referred patients (83%) met VF criteria (*p* = 0.521), demonstrating that VF loss was not associated with referral.

Legal blindness as defined in the USA was identified to impact eight of the 14 patients referred (57%): five patients based on their VA and three patients based on their VF. Five patients also qualified as having monocular status. Neither of these subcategories of vision loss was associated with a greater likelihood of referral for LVR services.

Thirteen out of the fourteen patients referred (93%) completed LVR visits. Five patients (38%) improved in their better-seeing eye after a low vision refraction; the average gain in BCVA was −0.18 ± 0.24 logMAR. Half of patients who achieved improvements in BCVA gained ≥ 2 lines: three in the better-seeing eye and one in the worse-seeing eye. In addition, 38% of patients received magnifiers and 15% mobility training. Of note, all patients who qualified as having low vision in our study were registered, as required, with the Massachusetts Commission for the Blind [25].

## 4. Discussion

Glaucoma can profoundly impact patient quality of life even at early stages when the degree of VF loss is mild [3]. Visual functioning and independence in modern society is especially hindered by loss of reading and driving capabilities [6]. Vision impairment and low vision can also lead to decreased emotional wellbeing or depression [14,26,27]. LVR services have been shown to help patients cope with visual impairment by improving their vision-related quality of life, psychological wellbeing, independence and mobility [18]. Despite compelling evidence of their effectiveness in achieving significant improvements in visual function at a reasonable cost [28,29], LVR services remain underutilized [30,31]. Our study adds to the growing body of research demonstrating a low rate of vision rehabilitation referral for persons with severe OAG and identifies factors associated with referrals. This study draws attention to VF loss as an additional factor that should prompt consideration of referral to LVR services for patients with OAG.

Determination of the need for LVR services was based on both the documented VA and VF measurements in our study, limited by the availability of HVF testing (93%). Not surprisingly, most patients who were referred for LVR services had low vision. However, we found that low vision status defined by VA was the factor most strongly associated with these referrals. Specifically, VA in the worse-seeing eye but not the better-seeing eye was associated with referral for LVR. Conversely, low vision status based on VF loss was not associated with referral for LVR services. This may stem from the fact that while VA can often be observed to improve with an updated refraction or by using various visual aides, VF loss may be perceived as less likely to benefit from LVR interventions. This might bias providers toward relying on VA limitations in clinical-decision making, even though studies show that LVR services can significantly improve patient mobility [5]. Our method of analyzing VFs allowed for identification of patients with VF of less than 20 degrees in the greatest VF diameter. Most patients who should have been considered for LVR referral based on VF were not referred (85%). In fact, according to the AAO Preferred Practice Patterns patients with *any* degree of VF loss can benefit from LVR services and should be considered for a referral [15].

Our analysis is restricted to the available quantitative data in the EHR. Kaleem et al. used a different approach—they carried out a survey among American Glaucoma Society members to assess LVR referral practices [21]. Respondents indicated that reports by patients of difficulties involving vision-related activities were the main driver for LVR referrals. Another prospective observational study, which examined traits of patients who attended LVR services offered at private outpatient clinics, reported that attendees were more commonly older females with macular disease and relatively preserved VA [32]. Interestingly, only 10% of patients were referred because of glaucoma. Owsley et al. had similar findings, namely that only 14% of U.S. patients accessing LVR were referred because of glaucoma-associated vision impairment [16]. At another large academic institution, 20% of low vision patients were found to utilize LVR over a longer 17-month study period [30]. In our study, most patients (88%) with severe OAG qualified as having low vision, yet fewer than 3% of those patients were referred for a low vision evaluation within the timeframe of the study. A cross-sectional study at a publicly funded eye clinic showed the referral rate to LVR for patients with irreversible, bilateral vision loss was nearly as low at 11%, with only 2% of those with unilateral vision impairment sent for LVR. In our study, around one quarter of patients with severe glaucoma were monocular, which may partially account for the low rate of referral we observed.

Importantly, since glaucoma is a progressive condition and patients referred for vision rehabilitation were often already functionally monocular, earlier referral to LVR could potentially have helped those patients cope with binocularity loss and visual function decrease over time [33]. For instance, one prospective observational study found 50% of LVR patients reported clinically meaningful improvement in visual abilities, measured as improvements in reading ability and emotional well-being [18,34]. Similar benefits are also reflected in our study by improvements in VA, as well as likely gains from mobility training and training in the use of optical devices. However, we lack any direct measures of functional outcomes or follow through with recommendations made by LVR specialists.

A few studies looked at the barriers to LVR referral and utilization and found that they exist at both physician and patient levels. Patients reported poor mental or physical health, transportation issues and lack of referrals as barriers to low vision rehabilitation. Providers, on the other hand, singled out poor overall health or cognitive function, older age, poor social support and low likelihood of follow-up [31]. Other studies identified physician-perceived economic restraints on the patient and short visit times as impediments [21]. Reassuringly, LVR referrals in our center did not depend on patient demographics or other factors including sex, race, ethnicity, primary language spoke, type of health insurance, employment status, distance from the eye clinic, glaucoma severity, or treatment characteristics. In our practice, all physicians were notified of the additional patients who could potentially benefit from the services but were not yet referred. It is our hope that this increased awareness will boost referral rates, but future studies are needed to assess these outcomes. In future, EHR-based clinical decision support systems could be used to facilitate LVR referrals [35].

The limitations of the present study include its modest sample size, retrospective nature and derivation from a suburban population based at an academic medical center. Conducting a study like ours in a population with greater racial and ethnic diversity would improve the generalizability of our results. Although we looked at patient demographic characteristics such as type of health insurance, distance from the eye clinic, and employment status, we did not directly take into account other barriers to care, such as access to transportation, level of education, or health literacy. A further limitation is that geographic data were available only at the ZIP code level. Moreover, our study was conducted during the second year of the COVID-19 pandemic. Older patients and those with disabilities may have been less likely to undergo eye examinations, including recommended LVR evaluations. Furthermore, a reduced availability of appointments caused by restrictions on access to routine health care services could have had an effect as well [36,37]. Telerehabilitation and mobile low vision services which were advanced during the pandemic may continue to help close these gaps [38,39,40]. Future studies should also seek to survey glaucoma providers and patients directly concerning their experience with low vision, barriers to accessing low vision care, as well as perceived benefits from LVR services delivered [17].

## 5. Conclusions

Despite a growing body of evidence that LVR services can improve visual function and enhance a patient’s vision-related quality of life, many patients with severe OAG do not receive referrals for those services. This gap in utilization not only affects the patients at our academic medical center but is a widespread phenomenon among glaucoma providers [30]. Our findings that most patients with severe OAG qualify as having low vision based upon both VA *and* VF criteria indicate that clinicians should use both of these domains to guide referrals for LVR services. Reassuringly, patients treated in our study showed meaningful improvements after utilizing these services. Because glaucoma is a progressive condition often leading to irreversible vision loss, the sooner patients access LVR services, the easier it is for them to learn to use and benefit from evolving assistive technologies. Increased efforts are needed to raise the rate at which LVR services are delivered so that more patients can benefit from these interventions.

## Figures and Tables

**Table 1:** Demographic and Clinical Characteristics of Patients with Severe Open-Angle Glaucoma.

CHARACTERISTIC	ALL (*n* = 522)	Group		*p* ^§^
		REFERRED (*n* = 14)	NOT REFERRED (*n* = 508)	
**Age, years**				
Median (IQR)	15.75, 79	87 (83.3 to 90.5)	79 (61 to 85)	
Range	35–100	65–94	35–100	**0.005**
**Sex, % (** ***n*** **)**				
Female	49.8 (260)	57.1 (8)	49.6 (252)	0.578
Male	50.2 (262)	42.9 (6)	50.4 (256)	
**Race and Ethnicity, % (** ***n*** **)**				
White (non-Hispanic)	83.7 (437)	85.7 (12)	83.7 (425)	0.837
Other races and ethnicities	16.3 (85)	14.3 (2)	16.3 (83)	
**Primary Language, % (** ***n*** **)**				
English	94.6 (494)	85.7 (12)	94.9 (482)	0.133
Other	5.4 (28)	14.3 (2)	5.1 (26)	
**Insurance Status, % (** ***n*** **)**				
Medicare	83.5 (436)	92.9 (12)	83.3 (423)	0.340
Medicaid	2.3 (12)	7.1 (1)	2.2 (11)	0.220
Commercial Insurance	11.9 (62)	—	12.2 (62)	0.164
Other Government	2.3 (12)	—	2.4 (12)	0.561
**Employment Status, % (** ***n*** **)**				
Working	19.0 (99)	0 (0)	16.7 (85)	0.094
Not working	81.0 (423)	100 (14)	83.3 (423)	
**Distance From clinic, miles**				
Mean (SD)	18.7 (29.5)	18.5 (28.7)	26.9 (51.4)	0.290
**Glaucoma Severity, % (** ***n*** **)**				
Severe OAG one eye	44.3 (231)	35.7 (5)	44.5 (226)	0.514
Severe OAG both eyes	55.7 (291)	64.3 (9)	55.5 (282)	
**Treatment Characteristics, % (** ***n*** **)**				
Incisional Surgery	49.2 (257)	42.9 (6)	49.4 (251)	0.629
Selective Laser Trabeculoplasty	35.2 (184)	28.6 (4)	35.4 (180)	0.596
Microinvasive Glaucoma Surgery	13.8 (72)	28.6 (4)	13.4 (68)	0.104
Cyclophotocoagulation	6.5 (34)	14.3 (2)	6.3 (32)	0.232
Any Surgery ^◊^	84.9 (443)	78.6 (11)	85.0 (432)	0.505
**Lens Status, % (** ***n*** **)**				
Phakic both eyes ^¶^	34.1 (178)	28.6 (4)	34.3 (174)	0.658
Pseudophakia both eyes	56.5 (295)	64.3 (9)	56.3 (286)	0.552
Pseudophakia, unilateral	9.4 (49)	7.1 (1)	9.4 (48)	0.770
**Visual Acuity, LogMAR**				
Better-seeing eye, mean (sd)	0.270 (0.378)	0.704 (0.365)	0.259 (0.586)	**<0.001**
Worse-Seeing Eye, mean (sd)	0.782 (0.860)	1.763 (0.836)	0.755 (1.128)	**<0.001**
**VF Testing, % (** ***n*** **) ^†^**				
Yes ^‡^	92.7 (484)	92.9 (13)	92.7 (471)	0.984
No	7.3 (38)	7.7 (1)	7.3 (37)	
**Low Vision Status, % (** ***n*** **)**				
Overall	88.1 (460)	92.9 (13)	85.6 (447)	0.579
Based on visual acuity	8.6 (42)	42.9 (6)	7.1 (36)	**<0.001**
Based on VF ^∞^	84.7 (442)	78.6 (11)	82.6 (431)	0.521
**Legal Blindness, % (** ***n*** **)**				
Overall	12.4 (65)	57.1 (8)	11.2 (57)	**<0.001**
Based on VA	7.1 (37)	42.9 (5)	6.1 (31)	**<0.001**
Based on VF ^¶^	6.9 (36)	21.4 (3)	6.5 (33)	0.101
**Monocular Status, % (** ***n*** **)**				
Yes	23.6 (123)	35.7 (5)	23.2 (118)	0.277
No	76.4 (399)	64.3 (9)	76.8 (390)	

^§^ Significance is marked in bold (*p* < 0.05). ^†^ Simulated binocular VF based on automated field testing. ^‡^ Patients for whom VF studies were available confirming the presence of severe open-angle glaucoma. ^◊^ Includes patients who had cataract extraction. ^∞^ Patients with any scotoma. ^¶^ Patients with VF equal or less than 20 degrees in the better-seeing eye.

**Table 2:** Individual Factors Associated with Referral of Patients with Severe Glaucoma to Low Vision.

Characteristic	aOR ^†^	95% CI		*p* ^§^
		Lower Bound	Upper Bound	
**VA**				
**Better-seeing Eye (LogMAR)**	1.76	0.68	4.57	0.245
**Worse-seeing Eye (LogMAR)**	2.03	1.08	3.80	**0.027**
**Low Vision Status**				
**Based on VA**	7.20	2.11	24.64	**<0.001**
**Based on VF**	0.90	0.24	3.37	0.876

^§^ Significance is marked in bold (*p* < 0.05). ^†^ aOR presented after adjusting for age.

## Data Availability

Due to the nature of this research, participants of this study did not agree for their data to be shared publicly, so supporting data are not available.

## Supplementary Materials

The following supporting information can be downloaded at: https://www.mdpi.com/article/10.3390/life16010012/s1, Figure S1: Strengthening the Reporting of Observational Studies in Epidemiology (STROBE) flow diagram.

## Abbreviations

The following abbreviations are used in this manuscript:

OAG	Neovascular age-related macular degeneration
CF	Counting fingers
LVR	Low vision rehabilitation
BCVA	Best-corrected visual acuity
OCT	Optical coherence tomography
VF	Visual field
