# Supplementary material for: Factors Associated with Referral to Low Vision for Patients with Advanced Glaucoma
Source: Life (Basel). 2025 Dec 22;16(1):12. doi: 10.3390/life16010012 (PMC12843145; doi:10.3390/life16010012)
Supplement: Supplementary file 1 [file life-16-00012-s001.zip › life-4000635-supplementary.pdf]

life

Supplementary Material

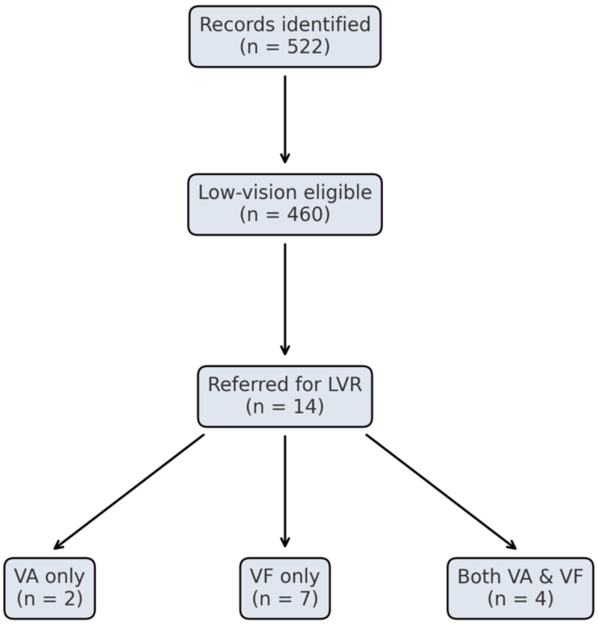

Figure S1. Strengthening the Reporting of Observational Studies in Epidemiology (STROBE) flow diagram.
